# Simultaneous Disulfide and Boronic Acid Ester Exchange in Dynamic Combinatorial Libraries

**DOI:** 10.3390/ijms160921858

**Published:** 2015-09-10

**Authors:** Sanna L. Diemer, Morten Kristensen, Brian Rasmussen, Sophie R. Beeren, Michael Pittelkow

**Affiliations:** 1 Department of Chemistry, University of Copenhagen, Universitetsparken 5, DK-2100 Copenhagen Ø, Denmark; E-Mails: sanna.lund_diemer@roche.com (S.L.D.); mortenfirst@gmail.com (M.K.); baru@novonordisk.com (B.R.); 2 Department of Chemistry, Technical University of Denmark, DK-2800 Kgs. Lyngby, Denmark

**Keywords:** dynamic combinatorial chemistry, supramolecular chemistry, disulfides, thiols, boronic acids, boronic acid esters, reversible chemical reactions

## Abstract

Dynamic combinatorial chemistry has emerged as a promising tool for the discovery of complex receptors in supramolecular chemistry. At the heart of dynamic combinatorial chemistry are the reversible reactions that enable the exchange of building blocks between library members in dynamic combinatorial libraries (DCLs) ensuring thermodynamic control over the system. If more than one reversible reaction operates in a single dynamic combinatorial library, the complexity of the system increases dramatically, and so does its possible applications. One can imagine two reversible reactions that operate simultaneously or two reversible reactions that operate independently. Both these scenarios have advantages and disadvantages. In this contribution, we show how disulfide exchange and boronic ester transesterification can function simultaneous in dynamic combinatorial libraries under appropriate conditions. We describe the detailed studies necessary to establish suitable reaction conditions and highlight the analytical techniques appropriate to study this type of system.

## 1. Introduction

Dynamic combinatorial chemistry is a supramolecular methodology that enables the discovery of specific and high affinity receptors [1]. Moreover, it can facilitate the thermodynamic synthesis of complex molecular architectures and the exploration of new binding motifs [2]. In dynamic combinatorial chemistry, a series of compounds are allowed to react using reversible chemistry (covalent or non-covalent) to give a mixture of compounds: a dynamic combinatorial library (DCL). DCLs are responsive to external stimuli such as the addition of a template, which, if recognized by one of more library members, can cause the DCL to adapt and alter its composition to minimize the overall free energy of the reaction mixture, often amplifying the binding-stabilized library member(s). 

The dynamic combinatorial approach relies on the availability of an arsenal of reversible chemical reactions [3,4]. In most studies exploring dynamic combinatorial libraries, a single type of reversible chemical reaction is used. Disulfide exchange [5], hydrazone exchange [6] and imine exchange [7] are amongst the most extensively utilized reactions in dynamic combinatorial chemistry. If two or more reversible reactions can be engineered into DCLs the structural diversity of the library increases dramatically [8,9,10,11,12,13,14,15,16,17,18,19,20]. In a DCL that makes use of two different reversible reactions, the reactions may be: (1) addressed simultaneously, where the reactions are reversible under the same reaction conditions; or (2) addressed orthogonally, where the two reactions are only reversible under different conditions. 

Examples of both simultaneous and orthogonal two-reaction DCLs have been described and both types of systems have attractive features. Most prominently, the orthogonal systems have been used by Leigh and coworkers in their molecular walker systems [21]. In that work, they take advantage of the orthogonal nature of the disulfide exchange reaction and the hydrazone exchange reaction. The disulfide exchange can be turned on under alkaline conditions where the hydrazone exchange does not occur, while hydrazone exchange can be turned on under acidic conditions where the disulfide exchange does not occur. This ability to control which reversible reaction is active at a given time enables the complex molecular walkers to function.

Simultaneous exchange of two or more reactions in DCLs has been explored using several different reversible reactions, and an advantage of this sort of system is the vast complexity and number of library members that are generated using only a small number of building blocks. This provides the system where the opportunities for identifying supramolecular receptors is larger due to the more effective exploration of chemical space examined in a single DCL [2].

Disulfide exchange [5] and boronic ester transesterification [22,23,24,25,26,27,28] are two of the most interesting reversible reactions for dynamic combinatorial chemistry. Both of these reactions have been explored independently under a variety of conditions in the context of dynamic combinatorial chemistry. Disulfide exchange has become the benchmark reversible reaction for DCLs in water, and a series of receptors for ammonium ions has been identified using this chemistry. A particularly elegant use of disulfide-based DCLs is in the identification of catalysts by recognition of transition state analogues of reactions [29,30,31]. Disulfide exchange chemistry has also been used in combination with other types of reversible chemical reactions. Besides the systems using disulfide exchange together with hydrazone exchange, described by Otto [11] and Furlan [12,13] and later used by Leigh [21], disulfide exchange has been used in combination with thioester exchange [13] and with imine exchange [14]. Disulfide exchange has also been explored in organic solvent, to identify receptors for electron deficient aromatic compounds using porphyrin-based systems [32,33]. Boronic ester transesterification too has been utilized in dynamic combinatorial chemistry both in water [28] where a novel approach to enzyme inhibition was developed and in organic solvents for the discovery of macrocycles and cages as receptors for small-molecules and ions [22,23,24,25,26,27]. Boronic ester transesterification has furthermore been explored in combination with imine exchange [18] and with reversible metal-ligand complex formation [16,17,18,19,20].

**Figure 1:**
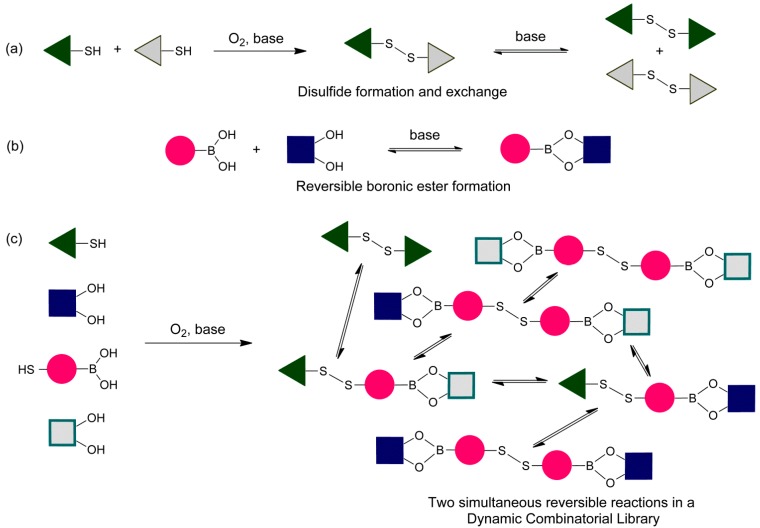
Reversible reaction used for dynamic combinatorial libraries in this work: (**a**) disulfide formation and exchange; (**b**) boronic acid ester formation; and (**c**) simultaneous disulfide and boronic acid ester formation and exchange.

With the aim of expanding the diversity in dynamic combinatorial libraries, we here introduce new double-level communicating DCLs in which reversible disulfide exchange and boronic ester transesterfication operate simultaneously (Figure 1). This type of DCL creates diversity in the library composition and also opens up for the possibility of selecting supramolecular receptors the recognize guests using not only the integral features of the building blocks themselves, but the functional groups that make up the connectivity [13].

## 2. Results and Discussion

**Figure 2:**
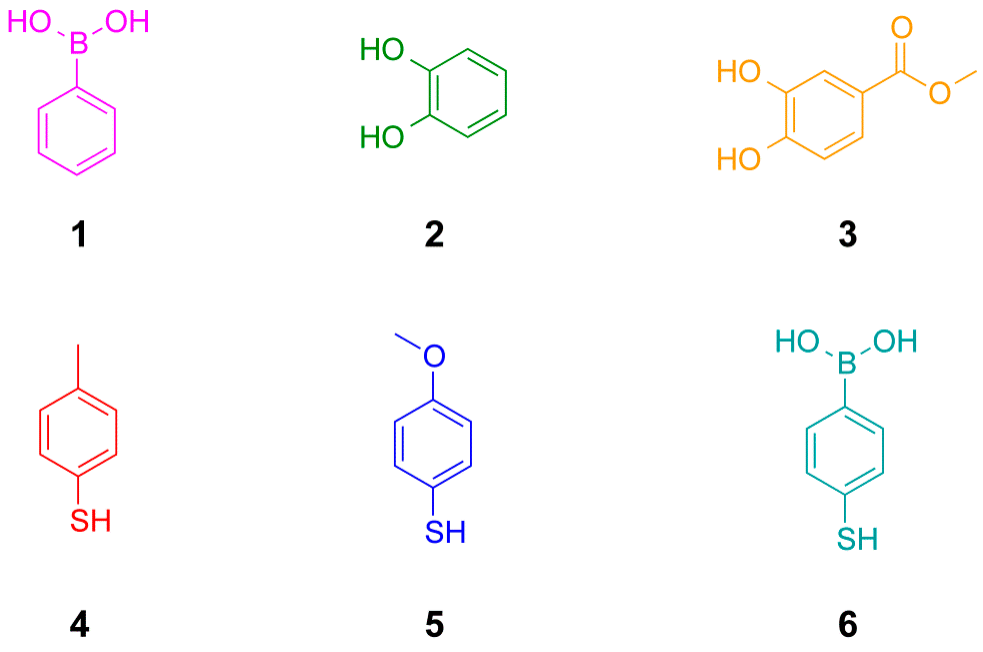
Structures of building blocks used in this study including phenylboronic acids, catechols (1,2-dihydroxy benzenes) and arylthiols.

We have chosen to use phenylboronic acids, catechols (1,2-dihydroxy benzenes) and aromatic thiols to develop the conditions for the simultaneous exchange of disulfide and boronic ester building blocks in dynamic combinatorial chemistry (Figure 2). We first examine the individual reactions and established conditions that ensure reversibility and that the libraries equilibrated under thermodynamic control (Figure 1). We then determine conditions compatible with both reactions. All experiments have been performed using CDCl_3_ as the solvent and a number of different experimental conditions have been examined with regards to other additives, different concentrations and bases.

### 2.1. Boronic Transesterification in CDCl_3_ Using Et_3_N as the Base

**Figure 3:**
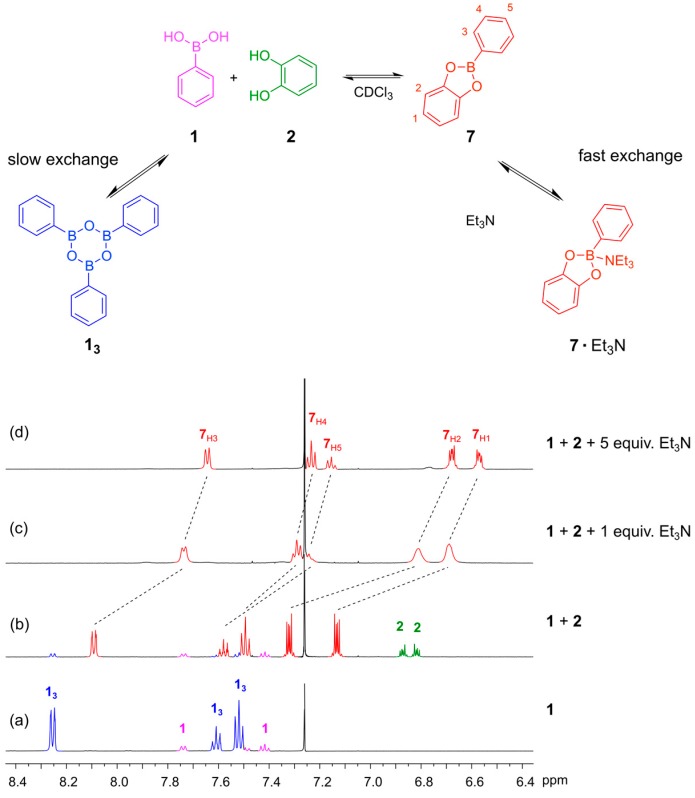
(**Top**) The equilibriums involved when mixing phenylboronic acid **1** with catechol **2** in CDCl_3_ in the presence and absence of Et_3_N; (**Bottom**) ^1^H-NMR spectra (500 MHz, CDCl_3_, 300 K): (**a**) Phenyl boronic acid **1** (10 mM). The spectrum shows that **1** and the corresponding trimeric boroxine **1_3_** are both present under these conditions; (**b**) The reaction mixture after 24 h when **1** and **2** (5 mM each) are mixed in the absence of base. The spectrum shows incomplete conversion to the boronic acid ester; (**c**) The reaction mixture after 24 h when **1**, **2** (4 mM each) and Et_3_N (4 mM) are combined in CDCl_3_. The spectrum shows broad feature indicating that the rate of exchange of the **7**·Et_3_N complex is comparable with the NMR chemical shift timescale; (**d**) The reaction mixture after 24 h when **1** (4 mM), **2** (4 mM) and Et_3_N (20 mM) are combined in CDCl_3_. The spectrum shows full conversion to the amine-bound boronic ester **7**·Et_3_N.

For the boronic ester exchange, conditions similar to those developed by Kubo *et al.* were examined [27], where phenylboronic acid and a catechol form the boronic acid ester. When recording the ^1^H-NMR spectra of the boronic acid **1** before adding the catechol, we observed both the phenylboronic acid and the corresponding boroxine trimer (**1_3_**) (Figure 3a). Triarylboraxines and boronic esters exist in equilibrium with each other, but the equilibrium is completely shifted towards the boronic acid ester in the presence of one equivalent of catechol and a suitable base. We studied the formation of the boronic ester of phenylboronic acid **1** and catechol **2** at 5 mM concentration. This reaction was fast and reached equilibrium within 10 min in CDCl_3_. In the absence of base, the boronic ester **7** is formed, but unreacted boronic acid and catechol are both still present, as seen in the ^1^H-NMR spectrum (Figure 3b). We proceeded to test how much base was required to push the equilibrium over to the ester. We first examined the use of Et_3_N, which has previously been used to promote disulfide exchange in organic solvent, and also tested DMAP (*N*,*N’*-4-dimethylamino pyridine) and pyridine, which gave similar results. When only one equivalent of Et_3_N was added, the ester **7** was formed but the ^1^H-NMR spectrum showed broad signals, suggesting that the exchange of the free and Et_3_N bound **7** occurs at an intermediate rate on the NMR timescale (Figure 3c). Upon addition of five equivalents of Et_3_N, nice well-defined signals were observed in the NMR spectrum, which is important considering that the objective in dynamic combinatorial chemistry is to generate complex mixtures of several compounds (Figure 3d).

**Figure 4:**
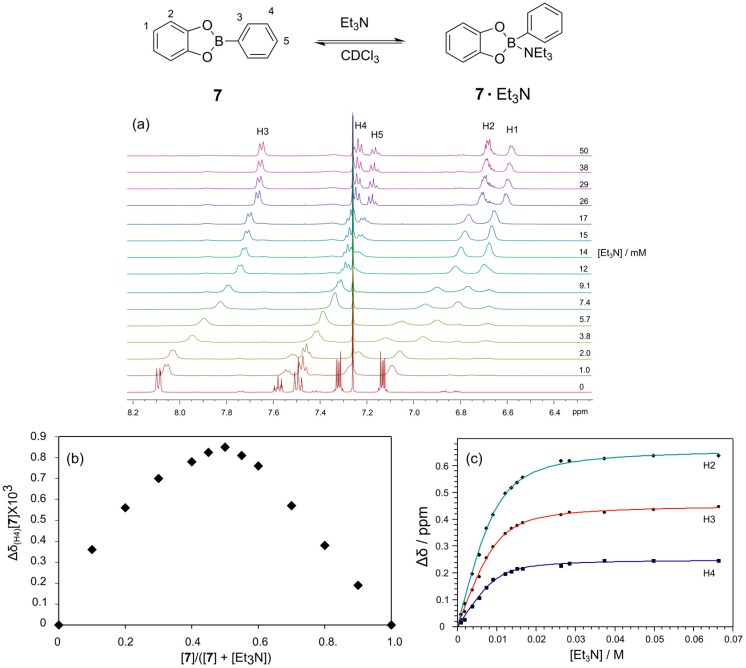
Determination of the binding strength and stoichiometry for the binding of Et_3_N to phenylboronic ester **7**. (**a**) ^1^H-NMR spectra (500 MHz, CDCl_3_, 300 K) of phenylboronic ester **7** (10 mM) in the presence of increasing concentrations of Et_3_N; (**b**) Job plot confirming the 1:1 binding stoichiometry for the interaction; (**c**) Plot of the chemical shift change of H3 of the phenylboronic ester **7** as a function of Et_3_N concentration and the non-linear fit to determine the binding constant.

To further understand the influence of the amine base, we studied the interaction of Et_3_N with the simple catechol based phenylboronic ester **7** using ^1^H-NMR spectroscopy (Figure 4). Upon titration of Et_3_N into a solution of **7** (10 mM) in CDCl_3_ all the aromatic peaks in the spectrum shifted upfield as a consequence of the binding of Et_3_N at the boron to give a tetrahedral geometry (Figure 4a). These changes in ^1^H-NMR chemical shift reflect a binding event that is fast on the NMR chemical shift time scale. We have established using a Job plot that Et_3_N binds to the boronic ester in a 1:1 fashion; the apex of the Job plot is at 0.5, as seen in Figure 4b. A plot of the chemical shift change of the meta proton on the boronic acid (H4) as a function of the Et_3_N concentration provides a binding isotherm from which a binding constant of 700 M^−1^ was determined (Figure 4c). Fitting of Binding Constants are provided in Supplementary Material.

### 2.2. Disulfide Exchange in CDCl_3_ Using Et_3_N as the Base

For the disulfide exchange reaction, we used conditions similar to those employed by Otto *et al.* [16] (using CH_3_CN as solvent), where dilute solutions of disulfides were equilibrated in the presence of a base. We wished to start our dynamic combinatorial libraries (DCLs) from free thiols, and therefore, in our experiments the thiols must first oxidize to the disulfides (an irreversible reaction) before the disulfide exchange can occur (a reversible reaction). However, for the disulfide exchange to proceed, a small amount of thiolate is required to mediate the reaction, so if the oxidation goes to completion too fast, the exchange reaction is unlikely to occur. This means that when starting a DCL from aryl thiols, a suitable solvent and a suitable base must be chosen to achieve reliable conditions which delicately balance the rate of oxidation and the rate of exchange to ensure the dynamic combinatorial library is under thermodynamic control.

**Figure 5:**
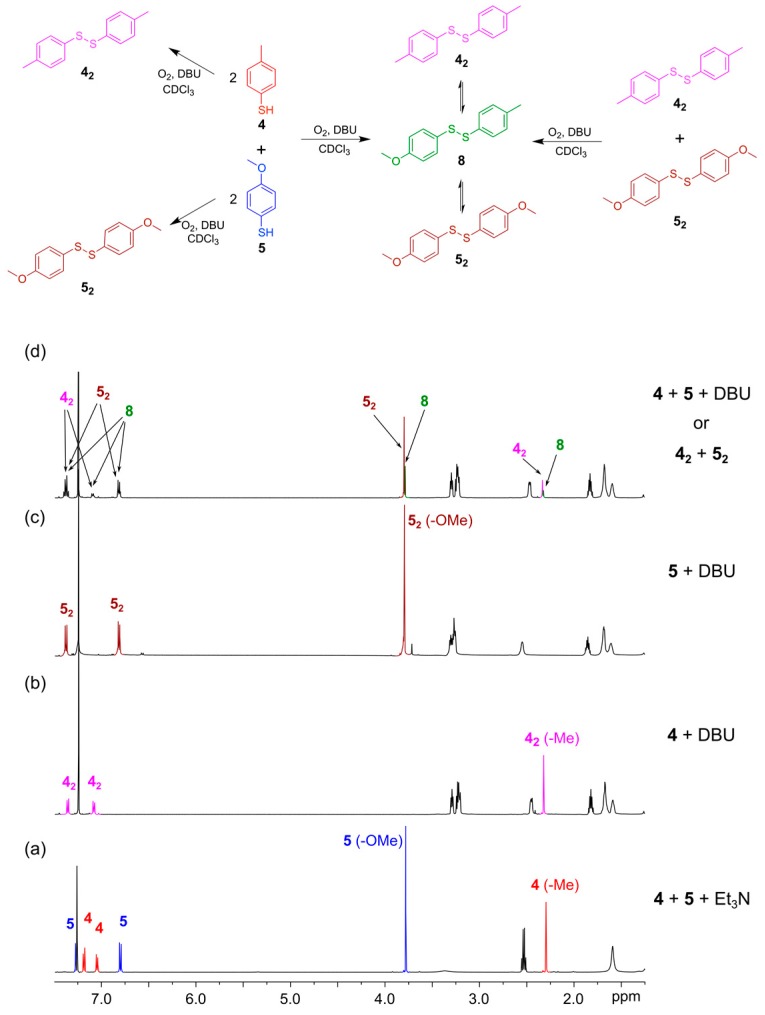
Establishing thermodynamic equilibrium with disulfides using DBU as the base in CDCl_3_. (**Top**) Reaction scheme illustrating how a DCL containing homo and heterodimers **4_2_**, **5_2_** and **8** can be formed either from the building blocks **4** and **5** or by mixing the *in situ* pre-formed dimers **4_2_** and **5_2_**; (**Bottom**) ^1^H-NMR spectra (500 MHz, CDCl_3_, 300 K): (**a**) The two thiols **4** (4 mM) and **5** (4 mM) after mixing in the presence of Et_3_N (20 mM) as the base. After 48 h only the thiols are present; (**b**) Disulfide **4_2_** (4 mM) generated in the presence of DBU (20 mM) *in situ*; (**c**) Disulfide **5_2_** (4 mM) generated in the presence of DBU (20 mM) *in situ*; (**d**) Equilibrium constitution achieved when either **4** and **5** (4 mM each) were reacted directly or equal volumes of solutions of *in situ* generated **4_2_** and **5_2_** (5 mM each) are combined in the presence of DBU (20 mM). Note the appearance of the heterodimer **8**.

In our hands, the oxidation of aryl thiols in CDCl_3_ in the presence of Et_3_N proceeded very slowly. A solution of 4-methylphenylthiol (**4**) (4 mM) and 5-methoxyphenylthiol (**5**) (4 mM) required weeks to form disulfides, even in the presence of a large excess of base (5 equivalent Et_3_N). Figure 5a shows the NMR spectrum of this solution after 48 h where only the free thiol starting materials are observed. This excessively slow oxidation of disulfides, which was confirmed by GC-MS is not practical for the formation of a dynamic combinatorial library.

### 2.3. Disulfide Exchange in CDCl_3_ Using DBU as the Base

To speed up this oxidation reaction, we tried a number of different bases. The more nucleophilic bases DMAP and pyridine were effective at facilitating the exchange of disulfides but the oxidation of thiols was still slow, requiring several days, and was therefore again not practical for DCLs. We reasoned that a stronger base could possibly help resolve this experimental problem. We searched for a base that would: (1) be stronger than Et_3_N; (2) be unreactive towards the substrates used; (3) be compatible with the wish to follow the process of the exchange reactions using ^1^H-NMR spectroscopy; (4) be transparent in the aromatic region of the ^1^H-NMR spectra; and (5) be simple to handle and non-toxic. With these requirements, we identified the stronger amidine base DBU (1,8-diazabicycloundec-7-ene) as a suitable candidate [32,33]. We proceeded to perform a series of experiments in which we determined that a disulfide DCLs could be generated from free thiols in CDCl_3_ in the presence of DBU. It was necessary to show that not only oxidation and disulfide formation occurred, but that there was also exchange of building blocks between disulfide library member takes place and that there was thermodynamic control over the DCL.

To validate that the disulfide exchange reaction is reversible and that a DCL operates under thermodynamic control, it must be possible to generate that DCL from different starting points. For example, if a DCL can be generated by mixing thiol building blocks, 4-methylphenylthiol (**4**) and 4-methoxyphenylthiol (**5**), it must also be possible to generate the same library composition by mixing the disulfides **4_2_** and **5_2_** in the presence of a catalytic amount of free thiol. To test this experimentally, we initiated the DCL from these two different starting points. In the first experiment, the DCL was initiated by mixing thiols **4** and **5** (each 4 mM) in CDCl_3_ with DBU (20 mM) in a NMR tube. The ^1^H-NMR spectrum was recorded regularly over three days. In the second experiment, the aryl thiols **4** and **5** (each 8 mM) were individually dissolved in two different NMR tubes with DBU (20 mM) added. After 24 h, the NMR spectrum of each sample was recorded showing the formation of homodimers **4_2_** and **5_2_** (Figure 5b,c). The two samples were then mixed in equal portions to form a second DCL and the ^1^H-NMR spectrum of the mixture was recorded regularly during the following three days. After one day, the NMR spectra of the two DCLs showed identical mixtures containing the homodimers, **4_2_** and **5_2_**, as well as the heterodimer **8**, indicating that exchange of building blocks took place and the libraries had reached equilibrium (Figure 5d). A GC/MS analysis of the two samples verified the formation of the homo- and hetero-disulfides. Disulfide Exchange is provided in Supplementary Material.

### 2.4. Boronic Transesterfication and Disulfide Exchange in CDCl_3_ Using DBU as the Base

**Figure 6:**
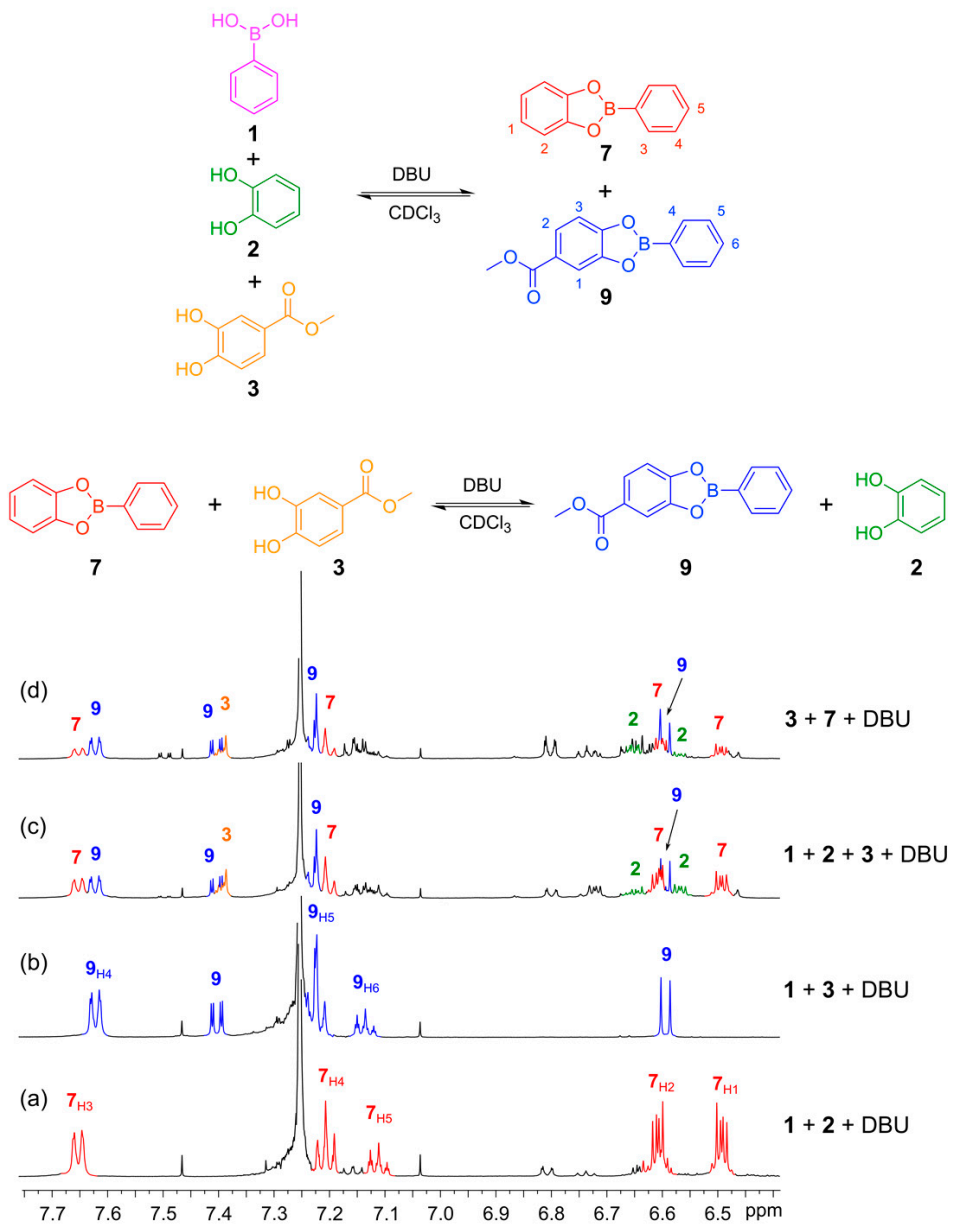
Establishing thermodynamic equilibrium with boronic ester transesterification using DBU as the base in CDCl_3_. (**Top**) Reaction schemes illustrating how boronic esters **7** and **9** can be formed either from their constituent building blocks **1**, **2** and **3** or by reacting the preformed boronic ester **7** with catechol **3** to give an equilibrium mixture containing esters **7** and **9** and catechols **2** and **3**; (**Bottom**) ^1^H-NMR spectra (500 MHz, CDCl_3_, 300 K): (**a**) *in situ*-generated phenylboronic ester **7** formed from **1** (4 mM) and **2** (4 mM) in the presence of DBU (20 mM) after 30 min; (**b**) *In situ*-generated phenylboronic ester **9** formed from **1** (4 mM) and **3** (4 mM) in the presence of DBU (20 mM) after 30 min; (**c**) DCL formed from **1** (2 mM), **2** (2 mM) and **3** (2 mM); (**d**) DCL formed from **7** (2 mM) and **3** (2 mM). Note that the spectra in c and d show the same composition. There are catechol decomposition products seen at 6.8 and 7.2 ppm. These are formed because the experiments are performed with a 1:1:1 ration of **1**, **2** and **7** so there is an excess of catechol.

With conditions in place to perform the disulfide exchange under thermodynamic control, we proceeded to verify the reversible nature of the boronic ester transesterification using DBU as the base. First, we verified that DBU mediated the formation of the boronic esters from phenyl boronic acid (**1**) with catechol (**2**) and methyl 3,4-dihydroxybenzoate (**3**). Five equivalents of DBU were utilized and boronic esters **7** and **9** were formed quantitatively giving well-defined ^1^H-NMR spectra with sharp features recorded after 30 min reaction time (Figure 6a,b). Next, we tested for reversibility and thermodynamic control over the system by initiating DCLs from different starting points. Figure 6c shows a DCL formed from the mixing of phenylboronic acid (**1**) with catechols **2** and **3** in a 1:1:1 ratio to give a mixture containing the two boronic esters, **7** and **9**, as well as excess of catechols **2** and **3**. Figure 6d shows the DCL formed by mixing the *in situ* formed boronic ester **7** (seen in spectrum 6a) with methyl 3,4-dihydroxybenzoate (**3**). The two spectra (Figure 6c,d) exhibit similar features, indicating that reversible transesterification proceeds and the mixture is under thermodynamic control. Establishing Equilibrium for Boronic Ester Transesterification is provided in Supplementary Material.

**Figure 7:**
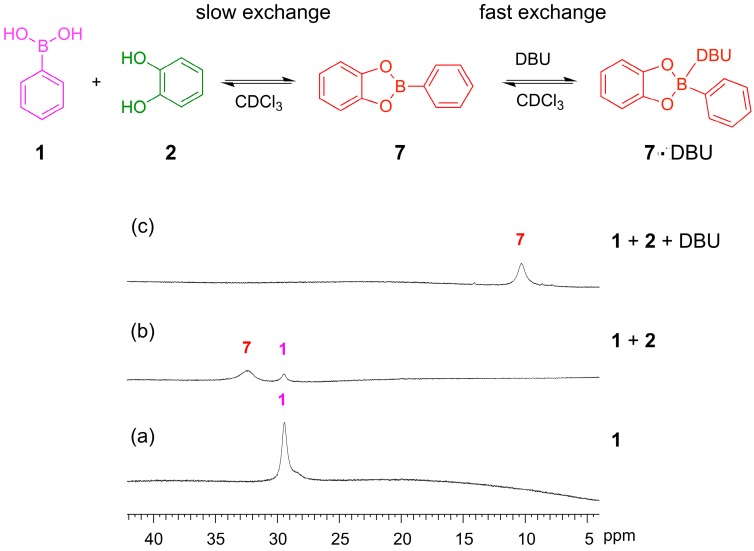
^11^B NMR. (160 MHz, CDCl_3_, 300 K) illustrating boronic ester formation and binding of DBU: (**a**) Phenylboronic acid **1** (10 mM); (**b**) Phenylboronic acid **1** (3.3 mM) and catechol **2** (3.3 mM) recorded after 24 h and showing the incomplete formation of the phenylboronic ester **7** with residual phenylboronic acid **1**; (**c**) **1** (3.3 mM), **2** (3.3 mM) and DBU (16.7 mM) recorded after 24 h and showing the formation of the tetrahedral boronate complex with DBU.

^11^B-NMR spectroscopy provides a useful tool to monitor the formation of boronic esters and study coordination at the boron center. We used ^11^B-NMR spectroscopy to observe the formation of boronic ester **7** from its constituent building blocks **1** and **2** and to examine the role of DBU in mediating the reaction. Upon addition of catechol **2** to a solution of phenylboronic acid **1**, a new signal is observed at 32 ppm, which can be assigned to the boronic ester **7** while some of boronic acid **1** is still present, illustrating the incomplete formation of the ester in the absence of base (Figure 7a,b). When five equivalents of DBU were added, the signal for the boronic acid **1** disappears and the signal for the ester **7** increases in size and shifts upfield to 10 ppm (Figure 7c). DBU binds to the boronic ester **7**, stabilizing this product, and thus mediates the complete conversion from the acid to the boronic ester. The upfield shift upon base binding is consistent with a change in hybridization of the boron center from trigonal planar to tetrahedral, as reported previously in the literature [34]. This result is similar to our previous observation that Et_3_N binds to boronic ester **7**.

**Figure 8:**
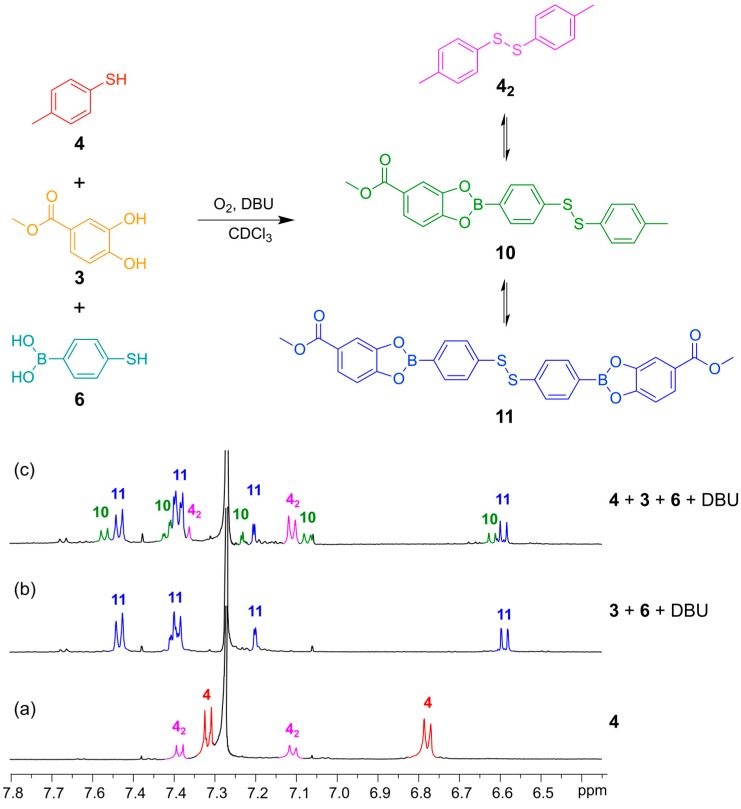
(**Top**) Simultaneous disulfide exchange and boronic ester transesterification combining two different thiol building blocks; (**Bottom**) ^1^H-NMR spectra (500 MHz, CDCl_3_, 300 K): (**a**) Thiol **4** (contains minor amounts of the disulfide **4_2_**); (**b**) Bifunctional building block **6** (4 mM) reacted with catechol **3** (4 mM) in the presence of DBU (20 mM); (**c**) Bifunctional building block **6** (2 mM) reacted with catechol **3** (2 mM) and thiol **4** (2 mM) in the presence of DBU (20 mM). All spectra recorded after 24 h reaction time.

**Figure 9:**
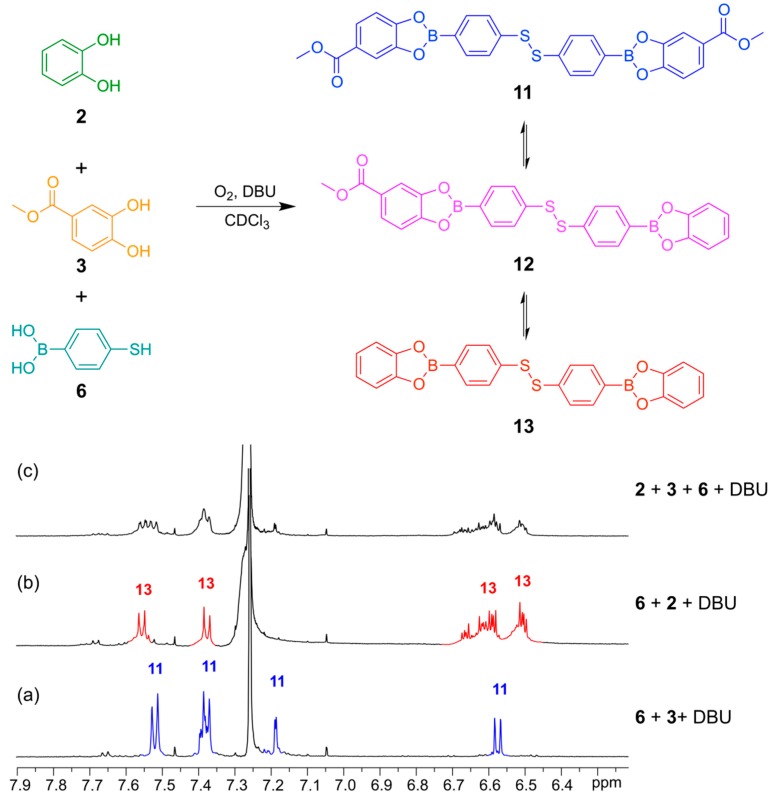
(**Top**) Simultaneous disulfide exchange and boronic ester transesterification combining two different diol building blocks; (**Bottom**) ^1^H-NMR spectra (500 MHz, CDCl_3_, 300 K): (**a**) Bifunctional building block **6** (4 mM) reacted with catechol **3** (4 mM) in the presence of DBU (20 mM); (**b**) Bifunctional building block **6** (4 mM) reacted with catechol **2** (4 mM) in the presence of DBU (50 Mm); (**c**) Bifunctional building block **6** (4 mM) reacted with catechol **2** (2 mM) and catechol **3** (2 mM) in the presence of DBU (20 mM). All spectra recorded after 24 h reaction time.

With proof of both reactions equilibrating under thermodynamic control using identical conditions (CDCl_3_, 5 equivalents DBU), we continued to test whether the two reversible reactions function simultaneously. This was confirmed according to the experiment outlined in Figure 8 and Figure 9. In both experiments, we use the bifunctional building block **6**, which contains both a boronic acid functionality and a thiol functionality.

In Figure 8 is shown ^1^H-NMR spectra that illustrate how the bifunctional building block **6** can engage in DCLs with both another thiol (4-methylphenylthiol (**4**)) and a catechol (methyl 3,4-dihydroxybenzoate (**3**)). A DCL containing the symmetrical homo-disulfides, **4_2_** and **11**, and the hetero-disulfide **10** formed within 24 h and the composition of this DCL was independent of the order of addition of the building blocks (Figure 8c).

In Figure 9 is illustrated how two different catechols (**2** and **3**) combine with thiol-functionalized phenyl boronic acid **6** to gives the three possible linear disulfide oligomers **11**, **12** and **13**. Once again, the same distribution of library members in the DCL was obtained after 24 h reaction time regardless of the order of addition of the building blocks.

## 3. Experimental Section 

All chemicals and solvents, unless otherwise stated, were purchased from commercial suppliers and used as received. NMR spectra were recorded on a Bruker Ultrashield Plus 500 MHz spectrometer (Copenhagen, Denmark). ^1^H-NMR, ^13^C-NMR and ^11^B-NMR spectra were recorded at 500 MHz, 125 MHz and 160 MHz, respectively, using residual non-deuterated solvent as the internal standard. Samples were prepared using CDCl_3_ purchased from Euriso-Top (Copenhagen, Denmark) or Cambridge Isotope Labs. The NMR data was processed using MestReNova v. 8.0.2. Assignment of all ^1^H and ^13^C resonances was achieved using standard 2D NMR techniques as ^1^H-^1^H COSY, ^1^H-^13^C HSQC, and ^1^H-^13^C HMBC. ^11^B-NMR were recorded at 160 MHz. Gas chromatography mass spectrometry (GC-MS) (Agilent Technologies, Santa Clara, CA, USA) was recorded on an Agilent 6890 Series GC-system combined with an Agilent 5973N mass selective detector.

## 4. Conclusions 

In conclusion, we have described conditions that allow disulfide and boronic transesterfication to be simultaneously addressed in a single dynamic combinatorial library. Aryl thiols, phenylboronic acids, and catechols are combined with building blocks equipped with both an aryl thiol and a boronic acid in the presence of DBU to generate DCLs at equilibrium within 24 h at room temperature. We have shown how a combination of mass spectrometry, ^1^H-NMR and ^11^B-NMR spectroscopy can be utilized to monitor these systems and test for the reversibility of the reactions and the thermodynamic control of the chemical system. Here, we have established the conditions using DCLs with linear constituents in organic solvent. Future work will expand these systems to incorporate tetra alcohols, dithiols and diboronic acids capable of generating macrocycles and capsules. Furthermore, we will explore building block designs and develop reaction conditions to enable the generation of these double-level DCLs in aqueous solution such that the synthesis of receptors and ligands for biological targets may be considered.

## Supplementary Materials

Supplementary materials describing the procedures for establishing equilibria and determining binding constants can be found at http://www.mdpi.com/1422-0067/16/09/21858/s1.
