# Supplementary material for: Simultaneous Disulfide and Boronic Acid Ester Exchange in Dynamic Combinatorial Libraries
Source: Int J Mol Sci. 2015 Sep 10;16(9):21858–72. doi: 10.3390/ijms160921858 (PMC4613285; doi:10.3390/ijms160921858)
Supplement: Supplementary file 1 [file ijms-16-21858-s001.pdf]

Supplementary Information

1. NMR Experiments for Disulfide Exchange and Boronic Ester Transesterification in $CDCl_3$

All building block samples were 10 mM in $CDCl_3$ and base concentration 100 mM in $CDCl_3$. All Boronic ester experiments were equilibrated for 30 min before equilibrium was reached and all disulfide samples were equilibrated for 20 h.

1.1. Disulfide Exchange (Figure 5 in Manuscript)

The reversibility was investigated by starting the reaction in two different ways.

Method (a): The NMR sample was prepared by mixing the two thiols immediately 1:1 with 5 equivalent. base. The NMR sample was left for 24 min before the sample was analyzed.

Method (b): The two NMR samples containing either thiol was prepared by mixing the individual thiol with 5 equivalent base. The NMR sample was left for 24 h before the sample was analyzed. Then, the two samples were combined and analyzed again after 24 h to confirm that equilibrium had been reached.

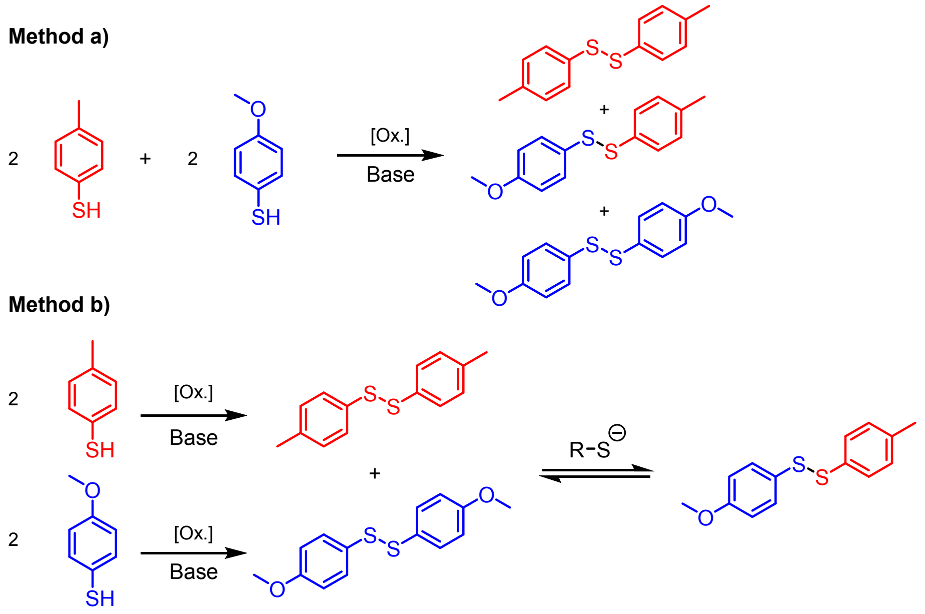

Figure S1. Schematic representation of the experimentsused to establish the thermodynamic equilibrium.

1.2. Establishing Equilibrium for Boronic Ester Transesterification (Figure 6 in Manuscript)

The reversibility was investigated by starting the reaction in two different ways.

Method (i): The NMR sample contained 2 equivalent phenylboronic acid (**1**), 1 equivalent catechol (**2**), 1 equivalent methyl 3,4-dihydroxybenzoate (**3**) and 5 equivalent base. The NMR sample was left for 30 minutes before the sample was analyzed.

Method (ii): The NMR sample contained 2 equivalent phenylboronic acid (**1**), 1 equivalent catechol (**2**) and 5 equivalent base. The NMR sample stood for 30 min. Then, 1 equivalent methyl

3,4-dihydroxybenzoate (**3**) was added and the sample was left for 30 min before analysis. In method (ii), the reverse order of addition was also investigated.

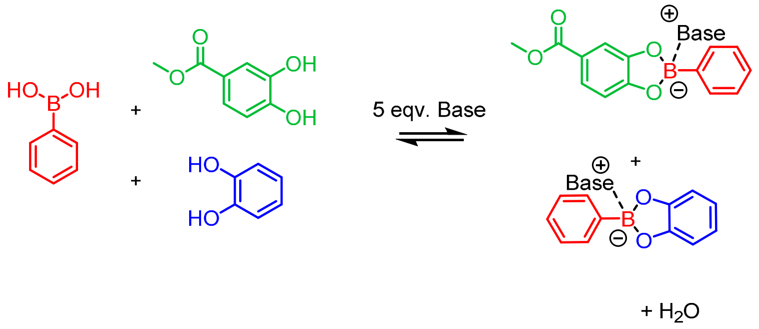

Figure S2. Schematic representation of the experimentsused to establish the thermodynamic equilibrium.

2. Fitting of Binding Constants (Figure 4 in Manuscript)

From the Job plots, a 1:1 stoichiometry between host and guest was found. Hence, the equilibrium constant for the host-guest complexation is given by Equation (1). In this expression, the denominator is expanded by substitution with $[H] = [H]_0 - [HG]$ and $[G] = [G]_0 - [HG]$, giving Equation (2).

$$K = \frac{[HG]}{[H][G]} \tag{1}$$

$$K = \frac{[HG]}{([H]_0 - [HG])([G]_0 - [HG])} = \frac{[HG]}{[H]_0[G]_0 - [HG]([H]_0 + [G]_0) + [HG]^2} \tag{2}$$

Equation (2) can be rearranged to the second order equation (Equation (3)) with $[HG]$ as the unknown, and the general solution is given in Equation (4).

$$0 = [HG]^2 - [HG]\left([G]_0 + [H]_0 + \frac{1}{K}\right) + [H]_0[G]_0 \tag{3}$$

$$[HG] = \frac{1}{2}\left(\left([G]_0 + [H]_0 + \frac{1}{K}\right) \pm \sqrt{\left([G]_0 + [H]_0 + \frac{1}{K}\right)^2 - 4[G]_0[H]_0}\right) \tag{4}$$

Only the solution where the last term is subtracted is chemically meaningful because the solution with a plus sign results in a concentration of complex that is higher than the smallest of the numbers $[G]_0$ and $[H]_0$.

Equation (4) gives an expression where the unknowns are $[HG]$ and K. The purpose is to find K and ^{1}H-NMR was used to provide a measure of $[HG]$. Various amounts of Et_3N were titrated into a solution of the boronic ester **7** under conditions where the total concentration of host was constant and the movement of a host signal (denoted δ) was followed.

Under the used conditions, the complexation was fast on the chemical shift time scale, and therefore the observed signal δ is as a weighted average of the signals δ_H (chemical shift of the proton in pure host) and δ_{HG} (chemical shift of the proton in pure complex) with the mole fractions X_H and X_{HG} as the

weighting factors. This is expressed in Equation (5), which, via standard manipulations, can be written as Equation (6).

$$\delta = \delta_H X_H + \delta_{HG} X_{HG}$$

$$= \delta_H \frac{[H]_o - [HG]}{[H]_o} + \delta_{HG} \frac{[HG]}{[H]_o} \quad (5)$$

$$= \delta_H + (\delta_{HG} - \delta_H) \frac{[HG]}{[H]_o} \quad (6)$$

For each measurement in the titration, the change from δ_H to the observed δ was calculated and denoted $\Delta\delta = \delta - \delta_H$. The unknown quantity, $\delta_{HG} - \delta_H$, indicates the maximal obtainable change in the titration and is denoted $\Delta\delta_{\mathrm{max}}$. With these notations, Equation (6) can be rewritten as Equation (7) and by substitution of Equation (4) into Equation (7), the final fitting equation, Equation (8), is obtained.

$$\Delta\delta = \Delta\delta_{\mathrm{max}} \frac{[HG]}{[H]_o} \quad (7)$$

$$= \frac{\Delta\delta_{\mathrm{max}}}{2[H]_0} \left(\left([G]_0 + [H]_0 + \frac{1}{K} \right) - \sqrt{\left([G]_0 + [H]_0 + \frac{1}{K} \right)^2 - 4[G]_0[H]_0} \right) \quad (8)$$

In Equation (8), the quantities $\Delta\delta_{\mathrm{max}}$ and K are unknown but linked to the measurable quantity Δδ and the known $[H]_0$ and $[G]_0$. Using the software, SciDavis, $\Delta\delta_{\mathrm{max}}$ and K were determined by fitting the equation data to Equation (8).

The chemical shift changes for H2, H3 and H4 were each monitored and used to determine Ddmax and K and the average taken.

Table S1. Binding constants determined.

H2	K	590 ± 50
	$\Delta\delta_{\mathrm{max}}$	0.67 ± 0.007
H3	K	690 ± 70
	$\Delta\delta_{\mathrm{max}}$	0.46 ± 0.005
H4	K	850 ± 130
	$\Delta\delta_{\mathrm{max}}$	0.25 ± 0.004
